# Bis(methyl xanthato)-κ*S*;κ^2^ *S*:*S*′-(triphenyl­phosphane-κ*P*)palladium(II)

**DOI:** 10.1107/S1600536811040487

**Published:** 2011-10-12

**Authors:** Reyna Reyes-Martínez, Simón Hernández-Ortega, David Morales-Morales

**Affiliations:** a Instituto de Química, Universidad Nacional Autónoma de México, Circuito Exterior, Ciudad Universitaria, México, D. F., 04510, Mexico

## Abstract

The title compound, [Pd(C_2_H_3_OS_2_)_2_(C_18_H_15_P)], features a palladium complex with a triphenyl­phosphane ligand and two xanthate ligands, one of them coordinates in a bidentate and the other in a monodentate fashion, giving rise to a slightly distorted square-planar coordination of the Pd^II^ ion. As a result of this difference in the coordination modes, the C—S bond lengths are different, *viz*. 1.687 (2) and 1.692 (2) Å in the bidentate ligand and 1.723 (2) Å in the monodentate ligand, whereas the non-coordinating S atom has a C—S distance of 1.649 (2) Å. The crystal packing is stabilized by C—H⋯O inter­actions.

## Related literature

For background information on xanthates, see: Karlin (2005 ▶); Friebolin *et al.* (2005 ▶). For crystal engineering, see: Tiekink (2003 ▶). For bond-length data, see: Allen (2002 ▶).
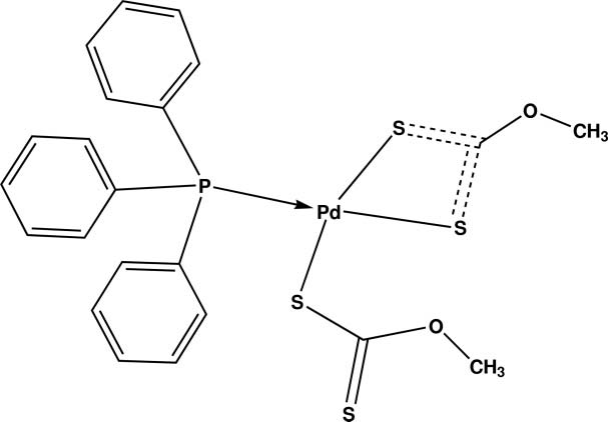


## Experimental

### 

#### Crystal data

[Pd(C_2_H_3_OS_2_)_2_(C_18_H_15_P)]
*M* *_r_* = 583.00
Triclinic, 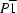

*a* = 9.5595 (10) Å
*b* = 9.5883 (10) Å
*c* = 14.4661 (16) Å
α = 73.619 (1)°
β = 87.492 (2)°
γ = 69.617 (1)°
*V* = 1190.3 (2) Å^3^
*Z* = 2
Mo *K*α radiation
μ = 1.22 mm^−1^
*T* = 298 K
0.30 × 0.22 × 0.20 mm

#### Data collection

Bruker SMART APEX CCD area-detector diffractometer
Absorption correction: analytical (*SADABS*, Bruker, 1999 ▶) *T*_min_ = 0.712, *T*_max_ = 0.817
9954 measured reflections
4371 independent reflections
4021 reflections with *I* > 2σ(*I*)
*R*_int_ = 0.025

#### Refinement

*R*[*F* ^2^ > 2σ(*F* ^2^)] = 0.025
*wR*(*F* ^2^) = 0.064
*S* = 1.10
4371 reflections
274 parameters
H-atom parameters constrained
Δρ_max_ = 0.32 e Å^−3^
Δρ_min_ = −0.42 e Å^−3^

### 

Data collection: *SMART* (Bruker, 1999 ▶); cell refinement: *SAINT* (Bruker, 1999 ▶); data reduction: *SAINT*; program(s) used to solve structure: *SHELXTL* (Sheldrick, 2008 ▶); program(s) used to refine structure: *SHELXTL*; molecular graphics: *XP* in *SHELXTL*; software used to prepare material for publication: *SHELXTL*.

## Supplementary Material

Crystal structure: contains datablock(s) I, global. DOI: 10.1107/S1600536811040487/bt5655sup1.cif

Structure factors: contains datablock(s) I. DOI: 10.1107/S1600536811040487/bt5655Isup2.hkl

Additional supplementary materials:  crystallographic information; 3D view; checkCIF report

## Figures and Tables

**Table 1:** Hydrogen-bond geometry (Å, °)

*D*—H⋯*A*	*D*—H	H⋯*A*	*D*⋯*A*	*D*—H⋯*A*
C18—H18⋯O20^i^	0.93	2.64	3.211	120

Symmetry code: (i) 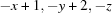.

**Fig. 1.**
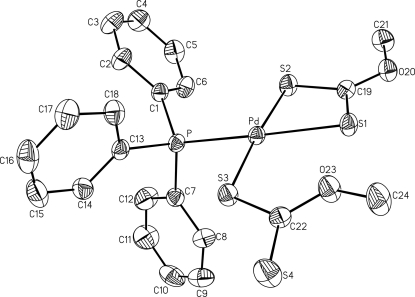
Crystal structure of the title compound with the numbering scheme. Displacement ellipsoids are shown at the 40% probability level. H atoms have been omitted for clarity.

## supplementary crystallographic information

### Comment

Xanthates ligands are a kind of compounds that can be coordinated to metal centers in different fashions, being found in coordination as monodentate, bidentate or even as a bridge, such richness in coordination modes very often leads to diverse structural motifs in the solid state (Karlin, 2005). It is precisely due to these structural features that these ligands are commonly involved in different supramolecular interactions such as D—H···S, D—H···O and S···Metal (D=C, N, O), all of these interactions being of important relevance to crystal engineering (Tiekink, 2003). Moreover, complexes including xanthate ligands in its structure have shown diverse applications at the industrial level, as chelating and flotation agents, while in bioinorganic chemistry they have found important applications as antitumoral agents (Friebolin *et al.* 2005).

The molecular structure of the title compound (I) shown in Figure 1, consists of two xanthate and one triphenylphosphane ligands coordinated to the Pd(II) center, in an almost square planar arrangement about the Pd(II) atom [0.0272 (3) Å]. The two xanthates ligands exhibiting different bond fashions, having one of them coordinated in a bidentate manner while the second one is attached to the palladium only by one sulfur atom in a monodentate way. The bond distances of Pd—S observed on the bidetate ligand 2.3386 (6)Å for Pd—S2 and 2.3581 (6)Å for Pd—S1 are larger than that observed Pd—S3 for the mondentate ligand. The difference between the above mentioned bond distances being due in part to the fact that the C22—S4 distance has a double bond character (1.649 (2) Å) and thus is shorter than C22—S4 (1.723 (2)Å which shows a commonly single bond character (Table 1). A revision of the Cambridge Structural Database (Allen, 2002), for Pd—S distances in bidentate and monodentate ligands, affords distance values of 2.31–2.33Å which are shorter than the data observed for the title compound (I). In absence of hydrogen bond donors, the molecules arrange as a centrosymmetric dimers generated by C—H···O and a weak intermolecular  Pd–S interaction of 3.521 (2)Å.

### Experimental

The title compound was synthesized by mixing Et_3_N (0.2 mL) and excess CS_2_ (2 mL) in methanol (10 mL). After stirring the resulting solution for 4 h at room temperature, *trans*-[(Ph_3_P)_2_PdCl_2_] (100 mg, 0.14 mmol) was added, affording a yellow precipitate that was filtered and washed with methanol. Crystals suitable for X-ray analysis were obtained upon recrystallization from a mixture of dichloromethane and isopropyl alcohol.

### Refinement

The positional parameters of H atoms were calculated geometrically (C—H = 0.93Å for C—H arom. and 0.96 for C—H of methyl groups). The H atoms were fixed with *U*_iso_(H) = 1.2Ueq and *U*_iso_(H) = 1.5Ueq of the attached non-H atom.

### Crystal data

**Table:**

[Pd(C_2_H_3_OS_2_)_2_(C_18_H_15_P)]	*Z* = 2
*M**_r_* = 583.00	*F*(000) = 588
Triclinic, *P*1	*D*_x_ = 1.627 Mg m^−^^3^
Hall symbol: -P 1	Mo *K*α radiation, λ = 0.71073 Å
*a* = 9.5595 (10) Å	Cell parameters from 8044 reflections
*b* = 9.5883 (10) Å	θ = 2.3–25.4°
*c* = 14.4661 (16) Å	µ = 1.22 mm^−^^1^
α = 73.619 (1)°	*T* = 298 K
β = 87.492 (2)°	Prism, orange
γ = 69.617 (1)°	0.30 × 0.22 × 0.20 mm
*V* = 1190.3 (2) Å^3^	

### Data collection

**Table:**

Bruker SMART APEX CCD area-detector diffractometer	4371 independent reflections
Radiation source: fine-focus sealed tube	4021 reflections with *I* > 2σ(*I*)
graphite	*R*_int_ = 0.025
Detector resolution: 0.83 pixels mm^-1^	θ_max_ = 25.4°, θ_min_ = 2.4°
ω scans	*h* = −11→11
Absorption correction: analytical (*SADABS*, Bruker, 1999)	*k* = −11→11
*T*_min_ = 0.712, *T*_max_ = 0.817	*l* = −17→17
9954 measured reflections	

### Refinement

**Table:**

Refinement on *F*^2^	Secondary atom site location: difference Fourier map
Least-squares matrix: full	Hydrogen site location: inferred from neighbouring sites
*R*[*F*^2^ > 2σ(*F*^2^)] = 0.025	H-atom parameters constrained
*wR*(*F*^2^) = 0.064	*w* = 1/[σ^2^(*F*_o_^2^) + (0.0336*P*)^2^ + 0.178*P*] where *P* = (*F*_o_^2^ + 2*F*_c_^2^)/3
*S* = 1.10	(Δ/σ)_max_ = 0.001
4371 reflections	Δρ_max_ = 0.32 e Å^−^^3^
274 parameters	Δρ_min_ = −0.42 e Å^−^^3^
0 restraints	Extinction correction: *SHELXTL* (Sheldrick, 2008), Fc^*^=kFc[1+0.001xFc^2^λ^3^/sin(2θ)]^-1/4^
Primary atom site location: structure-invariant direct methods	Extinction coefficient: 0.0063 (7)

### Special details

**Table:**

Geometry. All e.s.d.'s (except the e.s.d. in the dihedral angle between two l.s. planes) are estimated using the full covariance matrix. The cell e.s.d.'s are taken into account individually in the estimation of e.s.d.'s in distances, angles and torsion angles; correlations between e.s.d.'s in cell parameters are only used when they are defined by crystal symmetry. An approximate (isotropic) treatment of cell e.s.d.'s is used for estimating e.s.d.'s involving l.s. planes.
Refinement. Refinement of *F*^2^ against ALL reflections. The weighted *R*-factor *wR* and goodness of fit *S* are based on *F*^2^, conventional *R*-factors *R* are based on *F*, with *F* set to zero for negative *F*^2^. The threshold expression of *F*^2^ > σ(*F*^2^) is used only for calculating *R*-factors(gt) *etc*. and is not relevant to the choice of reflections for refinement. *R*-factors based on *F*^2^ are statistically about twice as large as those based on *F*, and *R*- factors based on ALL data will be even larger.

### Fractional atomic coordinates and isotropic or equivalent isotropic displacement parameters (Å^2^)

**Table:**

	*x*	*y*	*z*	*U*_iso_*/*U*_eq_	
Pd	0.557109 (17)	0.946630 (17)	0.158062 (11)	0.03533 (8)	
S1	0.62793 (7)	1.11131 (6)	0.02530 (4)	0.04484 (15)	
S2	0.77698 (7)	0.78738 (7)	0.10997 (4)	0.04619 (15)	
S3	0.34534 (7)	1.12011 (7)	0.20223 (4)	0.04483 (15)	
S4	0.16108 (9)	1.44896 (8)	0.16359 (6)	0.0674 (2)	
P	0.53719 (6)	0.75832 (6)	0.29129 (4)	0.03699 (14)	
C1	0.6235 (2)	0.5585 (2)	0.28557 (16)	0.0402 (5)	
C2	0.5382 (3)	0.4710 (3)	0.2782 (2)	0.0540 (6)	
H2	0.4346	0.5130	0.2779	0.065*	
C3	0.6064 (3)	0.3212 (3)	0.2714 (2)	0.0673 (8)	
H3	0.5482	0.2633	0.2661	0.081*	
C4	0.7577 (4)	0.2585 (3)	0.2723 (2)	0.0650 (8)	
H4	0.8027	0.1579	0.2676	0.078*	
C5	0.8448 (3)	0.3428 (3)	0.2801 (2)	0.0614 (7)	
H5	0.9484	0.2992	0.2811	0.074*	
C6	0.7775 (3)	0.4931 (3)	0.28646 (18)	0.0511 (6)	
H6	0.8364	0.5503	0.2914	0.061*	
C7	0.6282 (3)	0.7628 (3)	0.39762 (16)	0.0431 (5)	
C8	0.6358 (3)	0.9006 (3)	0.40261 (19)	0.0527 (6)	
H8	0.5990	0.9893	0.3507	0.063*	
C9	0.6979 (3)	0.9080 (4)	0.4844 (2)	0.0683 (8)	
H9	0.7010	1.0024	0.4876	0.082*	
C10	0.7548 (3)	0.7795 (4)	0.5605 (2)	0.0745 (9)	
H10	0.7967	0.7856	0.6154	0.089*	
C11	0.7498 (4)	0.6417 (4)	0.5555 (2)	0.0845 (10)	
H11	0.7905	0.5528	0.6067	0.101*	
C12	0.6853 (4)	0.6327 (3)	0.4754 (2)	0.0739 (9)	
H12	0.6801	0.5384	0.4735	0.089*	
C13	0.3460 (2)	0.7770 (2)	0.32140 (16)	0.0409 (5)	
C14	0.2973 (3)	0.7726 (3)	0.41336 (19)	0.0536 (6)	
H14	0.3635	0.7585	0.4632	0.064*	
C15	0.1497 (3)	0.7892 (4)	0.4307 (2)	0.0727 (9)	
H15	0.1165	0.7875	0.4923	0.087*	
C16	0.0524 (3)	0.8082 (4)	0.3579 (3)	0.0754 (9)	
H16	−0.0466	0.8190	0.3701	0.090*	
C17	0.1000 (3)	0.8112 (3)	0.2669 (2)	0.0656 (8)	
H17	0.0340	0.8219	0.2179	0.079*	
C18	0.2455 (3)	0.7983 (3)	0.24803 (18)	0.0521 (6)	
H18	0.2766	0.8039	0.1857	0.063*	
C19	0.7744 (2)	0.9505 (3)	0.02430 (16)	0.0398 (5)	
O20	0.87376 (17)	0.95924 (18)	−0.03937 (12)	0.0483 (4)	
C21	0.9931 (3)	0.8175 (3)	−0.0439 (2)	0.0579 (7)	
H21A	1.0437	0.7637	0.0187	0.087*	
H21B	1.0629	0.8431	−0.0894	0.087*	
H21C	0.9516	0.7522	−0.0641	0.087*	
C22	0.2955 (2)	1.3103 (3)	0.13247 (16)	0.0414 (5)	
O23	0.3687 (2)	1.33360 (19)	0.05373 (13)	0.0590 (5)	
C24	0.3206 (4)	1.4853 (3)	−0.0161 (2)	0.0767 (9)	
H24A	0.2189	1.5127	−0.0385	0.115*	
H24B	0.3835	1.4834	−0.0697	0.115*	
H24C	0.3276	1.5606	0.0137	0.115*	

### Atomic displacement parameters (Å^2^)

**Table:**

	*U*^11^	*U*^22^	*U*^33^	*U*^12^	*U*^13^	*U*^23^
Pd	0.03777 (12)	0.02838 (11)	0.03679 (11)	−0.01107 (8)	0.00156 (7)	−0.00519 (7)
S1	0.0442 (3)	0.0308 (3)	0.0494 (3)	−0.0087 (2)	0.0092 (3)	−0.0027 (2)
S2	0.0444 (3)	0.0313 (3)	0.0524 (3)	−0.0074 (2)	0.0049 (3)	−0.0038 (3)
S3	0.0462 (3)	0.0334 (3)	0.0465 (3)	−0.0086 (2)	0.0072 (3)	−0.0061 (2)
S4	0.0682 (5)	0.0440 (4)	0.0658 (4)	0.0038 (3)	0.0171 (4)	−0.0105 (3)
P	0.0415 (3)	0.0315 (3)	0.0367 (3)	−0.0151 (2)	−0.0002 (2)	−0.0046 (2)
C1	0.0460 (12)	0.0321 (11)	0.0396 (12)	−0.0144 (10)	0.0015 (9)	−0.0044 (9)
C2	0.0512 (14)	0.0393 (13)	0.0719 (17)	−0.0182 (11)	0.0005 (12)	−0.0130 (12)
C3	0.0733 (19)	0.0424 (15)	0.092 (2)	−0.0259 (14)	−0.0023 (16)	−0.0193 (15)
C4	0.079 (2)	0.0354 (14)	0.0713 (18)	−0.0102 (14)	0.0053 (15)	−0.0130 (13)
C5	0.0541 (15)	0.0444 (15)	0.0676 (17)	−0.0055 (12)	0.0035 (13)	−0.0033 (13)
C6	0.0478 (14)	0.0423 (14)	0.0576 (15)	−0.0166 (11)	−0.0018 (11)	−0.0038 (11)
C7	0.0448 (12)	0.0462 (13)	0.0390 (12)	−0.0183 (11)	0.0016 (9)	−0.0100 (10)
C8	0.0506 (14)	0.0484 (14)	0.0602 (15)	−0.0137 (12)	−0.0036 (12)	−0.0208 (12)
C9	0.0618 (17)	0.075 (2)	0.076 (2)	−0.0173 (15)	−0.0012 (15)	−0.0412 (17)
C10	0.0581 (17)	0.118 (3)	0.0530 (17)	−0.0248 (18)	0.0002 (13)	−0.0404 (19)
C11	0.113 (3)	0.084 (2)	0.0465 (16)	−0.032 (2)	−0.0208 (17)	−0.0012 (16)
C12	0.109 (2)	0.0577 (17)	0.0515 (16)	−0.0359 (18)	−0.0187 (16)	0.0022 (13)
C13	0.0447 (12)	0.0312 (11)	0.0446 (12)	−0.0152 (10)	0.0043 (10)	−0.0051 (9)
C14	0.0610 (16)	0.0489 (14)	0.0514 (14)	−0.0208 (13)	0.0104 (12)	−0.0142 (12)
C15	0.0672 (19)	0.075 (2)	0.0714 (19)	−0.0249 (16)	0.0293 (16)	−0.0181 (16)
C16	0.0468 (16)	0.069 (2)	0.099 (3)	−0.0192 (15)	0.0192 (17)	−0.0101 (18)
C17	0.0473 (15)	0.0649 (18)	0.075 (2)	−0.0190 (14)	−0.0063 (14)	−0.0050 (15)
C18	0.0465 (13)	0.0534 (15)	0.0492 (14)	−0.0169 (12)	0.0005 (11)	−0.0042 (12)
C19	0.0379 (11)	0.0373 (12)	0.0426 (12)	−0.0126 (10)	−0.0004 (9)	−0.0092 (10)
O20	0.0407 (9)	0.0436 (9)	0.0509 (9)	−0.0084 (7)	0.0091 (7)	−0.0080 (8)
C21	0.0419 (13)	0.0569 (16)	0.0669 (17)	−0.0056 (12)	0.0112 (12)	−0.0216 (14)
C22	0.0411 (12)	0.0374 (12)	0.0437 (12)	−0.0111 (10)	−0.0005 (10)	−0.0111 (10)
O23	0.0658 (11)	0.0359 (9)	0.0562 (10)	−0.0039 (8)	0.0164 (9)	−0.0036 (8)
C24	0.093 (2)	0.0384 (15)	0.0670 (19)	−0.0032 (15)	0.0225 (16)	0.0050 (13)

### Geometric parameters (Å, °)

**Table:**

Pd—P	2.2924 (6)	C9—H9	0.9300
Pd—S3	2.3267 (6)	C10—C11	1.361 (5)
Pd—S2	2.3386 (6)	C10—H10	0.9300
Pd—S1	2.3581 (6)	C11—C12	1.373 (4)
S1—C19	1.687 (2)	C11—H11	0.9300
S2—C19	1.692 (2)	C12—H12	0.9300
S3—C22	1.723 (2)	C13—C14	1.384 (3)
S4—C22	1.649 (2)	C13—C18	1.386 (3)
P—C7	1.818 (2)	C14—C15	1.384 (4)
P—C13	1.819 (2)	C14—H14	0.9300
P—C1	1.827 (2)	C15—C16	1.367 (5)
C1—C2	1.383 (3)	C15—H15	0.9300
C1—C6	1.384 (3)	C16—C17	1.369 (4)
C2—C3	1.386 (4)	C16—H16	0.9300
C2—H2	0.9300	C17—C18	1.375 (4)
C3—C4	1.359 (4)	C17—H17	0.9300
C3—H3	0.9300	C18—H18	0.9300
C4—C5	1.373 (4)	C19—O20	1.301 (3)
C4—H4	0.9300	O20—C21	1.455 (3)
C5—C6	1.387 (4)	C21—H21A	0.9600
C5—H5	0.9300	C21—H21B	0.9600
C6—H6	0.9300	C21—H21C	0.9600
C7—C8	1.371 (3)	C22—O23	1.316 (3)
C7—C12	1.381 (3)	O23—C24	1.447 (3)
C8—C9	1.377 (4)	C24—H24A	0.9600
C8—H8	0.9300	C24—H24B	0.9600
C9—C10	1.359 (4)	C24—H24C	0.9600
			
P—Pd—S3	88.08 (2)	C10—C11—C12	120.6 (3)
P—Pd—S2	95.32 (2)	C10—C11—H11	119.7
S3—Pd—S2	175.72 (2)	C12—C11—H11	119.7
P—Pd—S1	168.78 (2)	C11—C12—C7	120.3 (3)
S3—Pd—S1	101.78 (2)	C11—C12—H12	119.8
S2—Pd—S1	74.60 (2)	C7—C12—H12	119.8
C19—S1—Pd	85.06 (8)	C14—C13—C18	119.3 (2)
C19—S2—Pd	85.56 (8)	C14—C13—P	122.88 (19)
C22—S3—Pd	115.44 (8)	C18—C13—P	117.85 (18)
C7—P—C13	105.95 (10)	C15—C14—C13	119.7 (3)
C7—P—C1	104.63 (10)	C15—C14—H14	120.2
C13—P—C1	104.52 (10)	C13—C14—H14	120.2
C7—P—Pd	110.69 (8)	C16—C15—C14	120.4 (3)
C13—P—Pd	114.35 (7)	C16—C15—H15	119.8
C1—P—Pd	115.78 (7)	C14—C15—H15	119.8
C2—C1—C6	118.7 (2)	C15—C16—C17	120.3 (3)
C2—C1—P	121.55 (18)	C15—C16—H16	119.9
C6—C1—P	119.78 (18)	C17—C16—H16	119.9
C1—C2—C3	120.4 (2)	C16—C17—C18	120.0 (3)
C1—C2—H2	119.8	C16—C17—H17	120.0
C3—C2—H2	119.8	C18—C17—H17	120.0
C4—C3—C2	120.3 (3)	C17—C18—C13	120.3 (3)
C4—C3—H3	119.9	C17—C18—H18	119.8
C2—C3—H3	119.9	C13—C18—H18	119.8
C3—C4—C5	120.4 (3)	O20—C19—S1	119.69 (17)
C3—C4—H4	119.8	O20—C19—S2	125.54 (17)
C5—C4—H4	119.8	S1—C19—S2	114.77 (13)
C4—C5—C6	119.7 (3)	C19—O20—C21	118.96 (19)
C4—C5—H5	120.2	O20—C21—H21A	109.5
C6—C5—H5	120.2	O20—C21—H21B	109.5
C1—C6—C5	120.6 (2)	H21A—C21—H21B	109.5
C1—C6—H6	119.7	O20—C21—H21C	109.5
C5—C6—H6	119.7	H21A—C21—H21C	109.5
C8—C7—C12	118.7 (2)	H21B—C21—H21C	109.5
C8—C7—P	119.16 (18)	O23—C22—S4	123.92 (17)
C12—C7—P	122.1 (2)	O23—C22—S3	115.46 (16)
C7—C8—C9	120.1 (3)	S4—C22—S3	120.60 (14)
C7—C8—H8	120.0	C22—O23—C24	119.4 (2)
C9—C8—H8	120.0	O23—C24—H24A	109.5
C10—C9—C8	121.1 (3)	O23—C24—H24B	109.5
C10—C9—H9	119.5	H24A—C24—H24B	109.5
C8—C9—H9	119.5	O23—C24—H24C	109.5
C9—C10—C11	119.2 (3)	H24A—C24—H24C	109.5
C9—C10—H10	120.4	H24B—C24—H24C	109.5
C11—C10—H10	120.4		
			
P—Pd—S1—C19	26.14 (14)	C13—P—C7—C12	81.9 (3)
S3—Pd—S1—C19	177.19 (8)	C1—P—C7—C12	−28.2 (3)
S2—Pd—S1—C19	−0.47 (8)	Pd—P—C7—C12	−153.6 (2)
P—Pd—S2—C19	−174.52 (8)	C12—C7—C8—C9	−0.8 (4)
S3—Pd—S2—C19	−32.0 (3)	P—C7—C8—C9	176.9 (2)
S1—Pd—S2—C19	0.46 (8)	C7—C8—C9—C10	1.2 (4)
P—Pd—S3—C22	174.99 (9)	C8—C9—C10—C11	−0.1 (5)
S2—Pd—S3—C22	32.3 (3)	C9—C10—C11—C12	−1.4 (5)
S1—Pd—S3—C22	0.40 (9)	C10—C11—C12—C7	1.8 (6)
S3—Pd—P—C7	−85.16 (8)	C8—C7—C12—C11	−0.7 (5)
S2—Pd—P—C7	92.24 (8)	P—C7—C12—C11	−178.3 (3)
S1—Pd—P—C7	66.54 (14)	C7—P—C13—C14	−7.9 (2)
S3—Pd—P—C13	34.40 (8)	C1—P—C13—C14	102.3 (2)
S2—Pd—P—C13	−148.20 (8)	Pd—P—C13—C14	−130.10 (18)
S1—Pd—P—C13	−173.90 (12)	C7—P—C13—C18	171.35 (18)
S3—Pd—P—C1	156.01 (8)	C1—P—C13—C18	−78.4 (2)
S2—Pd—P—C1	−26.60 (8)	Pd—P—C13—C18	49.2 (2)
S1—Pd—P—C1	−52.30 (14)	C18—C13—C14—C15	0.1 (4)
C7—P—C1—C2	127.6 (2)	P—C13—C14—C15	179.3 (2)
C13—P—C1—C2	16.5 (2)	C13—C14—C15—C16	0.8 (4)
Pd—P—C1—C2	−110.26 (19)	C14—C15—C16—C17	−0.2 (5)
C7—P—C1—C6	−53.8 (2)	C15—C16—C17—C18	−1.3 (5)
C13—P—C1—C6	−164.93 (19)	C16—C17—C18—C13	2.2 (4)
Pd—P—C1—C6	68.3 (2)	C14—C13—C18—C17	−1.5 (4)
C6—C1—C2—C3	−0.4 (4)	P—C13—C18—C17	179.2 (2)
P—C1—C2—C3	178.2 (2)	Pd—S1—C19—O20	−179.27 (18)
C1—C2—C3—C4	0.3 (5)	Pd—S1—C19—S2	0.68 (11)
C2—C3—C4—C5	0.1 (5)	Pd—S2—C19—O20	179.3 (2)
C3—C4—C5—C6	−0.4 (5)	Pd—S2—C19—S1	−0.69 (11)
C2—C1—C6—C5	0.0 (4)	S1—C19—O20—C21	−175.44 (16)
P—C1—C6—C5	−178.6 (2)	S2—C19—O20—C21	4.6 (3)
C4—C5—C6—C1	0.4 (4)	Pd—S3—C22—O23	9.4 (2)
C13—P—C7—C8	−95.7 (2)	Pd—S3—C22—S4	−172.25 (10)
C1—P—C7—C8	154.22 (19)	S4—C22—O23—C24	−6.8 (4)
Pd—P—C7—C8	28.8 (2)	S3—C22—O23—C24	171.4 (2)

### Hydrogen-bond geometry (Å, °)

**Table:**

*D*—H···*A*	*D*—H	H···*A*	*D*···*A*	*D*—H···*A*
C18—H18···O20^i^	0.93	2.64	3.211	120.

Symmetry codes: (i) −*x*+1, −*y*+2, −*z*.
